# International comparison of health promotion at higher education institutions in Germany and the role of the German Prevention Act

**DOI:** 10.3389/fpubh.2023.1128556

**Published:** 2023-02-01

**Authors:** Pavel Dietz, Markus Schäfer

**Affiliations:** ^1^Institute of Occupational, Social and Environmental Medicine, University Medical Centre of the Johannes Gutenberg University, Mainz, Germany; ^2^Department of Communication, Johannes Gutenberg University, Mainz, Germany

**Keywords:** prevention law, health promotion, wellbeing, university, student, tertiary education, college

## 1. Introduction

The Ottawa Charter for Health Promotion in 1986 emphasized the importance of a supportive environment to enable people to increase control over and improve their health (1). Based on this charter, the Okanagan Charter in 2015 further emphasized the relevance of higher education institutions (HEIs) and their local and global influence on the development of individuals, societies, and cultures (2). It further highlighted the interdependence of the wellbeing of people, places, and our planet and concluded that HEIs are key settings of systemic health promotion since two lifeworlds^1^ meet here: the lifeworld of working and the lifeworld of studying. Furthermore, the Okanagan Charter builds the framework of an international network of health-promoting universities and colleges and provides a kind of guide on relevant aspects to becoming a health-promoting university or college. One central call to action is that HEIs should embed health into all aspects of campus culture, including its administration, operations, and academic mandates.

In 2015, the German parliament adopted “the act to strengthen health promotion and prevention” [Prevention Act (3)], which aims to strengthen health promotion in the lifeworlds and to foster cooperation between social security institutions and national and local governments. The act lists both lifeworlds represented at HEIs—the lifeworlds of working and studying—as particularly relevant for health promotion and prevention. According to the Prevention Act, the German salutary health insurances have to fund timely limited projects for health promotion in the lifeworlds. A central idea is to implement, with the help of these projects, health-promoting ideals permanently and sustainably in the structures and the self-image of the respective lifeworld (e.g., by developing health-promoting conditions and connecting relevant stakeholders), so that the structures remain in place beyond the funding period and empower the people in charge to carry out fundamental health-promoting tasks in future without the (financial) support of the salutary health insurance.

The present article aims to provide an expert's opinion^2^ on the status of health promotion at German HEIs in comparison to other countries. We will furthermore critically discuss the German act to strengthen health promotion and prevention, which has often been described as a health policy milestone, from a practical point of view (that of a health-promotion actor at a large German university). Finally, we will take a look into the future and outline some recommendations to support German HEIs on their way to becoming a health-promoting institution.

## 2. International comparison of health promotion at higher education institutions in Germany and the role of the German Prevention Act

The Okanagan Charter provides a solid framework for health-promoting universities and formulates, in particular, two calls-to-action for HEIs: First, to embed health into all aspects of campus culture; second, to lead health promotion action and collaboration locally and globally.

With regard to the first call, a health-promoting university should be understood as more than just an institution that conducts some health-promoting projects. It should further work toward the general mission to take health and health promotion in all decisions at HEIs into account, aspiring to create a learning environment and organizational culture that enhances the health of all the members of its community. To achieve this goal requires the explicit will, support, participation, commitment, and reliability of the respective institution leaders and executives (e.g., presidents, chancellors, rectors, and deans).

Internationally, HEIs are formally invited to adopt the Okanagan Charter by signing it, a way for institution leaders to strengthen and formalize their commitment to activating the Charter's vision, calls to action, and principles. The webpages of the local networks of health-promoting universities and colleges (6) list institutions that have already adopted the Charter or that are committed to the Charter's principles, vision, and aspirations. A closer look at these lists shows that, for example, in Canada, 42 HEIs have already adopted the Charter. In the United States of America 126 network campuses, in the United Kingdom 98 member institutions, and in Australia 25 network institutions are listed. Also, for Spain, a European country almost similar to Germany regarding size and population, already 61 member institutions are listed. In comparison, taking a closer look at the webpage of the German Network of Health Promoting Universities, it is not apparent how many and which HEIs are actually part of the German network. Furthermore, to the best of our knowledge, up to now, only two German HEIs have officially signed and adopted the Okanagan Charter. Most recently, this was the case in 2019, when the University of Applied Sciences for Public Administration of North Rhine Westphalia signed the charter.

In our opinion, these facts throw a spotlight on a fundamental problem with regard to health promotion at HEIs in Germany. At German HEIs, health promotion is very often limited to single and timely limited health-promoting projects, which are in most cases raised and conducted by individually motivated scientists or practical actors. It is more of a rarity that health promotion is initiated and permanently funded by the institution leaders in a top-down process. One might get the impression that university leaders as well as the state governments, who are responsible for framework legislation and funding for HEIs in Germany, unlike many areas of the private economy sector, often only consider the costs and not the benefits of a health-promoting institution. Consequently, since health promotion at HEIs very often does not go beyond project character due to failing to establish sustainable structures, HEIs in Germany are far away from being health-promoting institutions in the sense of the Okanagan Charter. Here, we see a large discrepancy with other countries. And in the medium to long run, a major problem of international competitiveness is the competition for excellence, students, and employees.

In principle, the idea to move institutions away from a project approach toward a more systemic or institutional approach is also anchored in the above-mentioned German Prevention Act, a German federal law. It is an explicit goal of the act to implement projects that are funded as part of the Prevention Act after the maximum funding period of 5 years into the permanent structures of the respective institutions. With a focus on HEIs, this would mean that, after a positive evaluation, health-promoting actions have to be financed by the institution's management or specific departments.

Theoretically, this is a good idea. The start-up funding allows public institutions like German HEIs to set up and try out appropriate structures without having to take big financial risks. But, in reality, given the way things are going at the moment at HEIs, the ultimate goals of the law and funding are not even close to being achieved, due to overcoming the mindset and logic of the higher education system and its lack of sustainable financing by German politics. As a consequence, a lot of money is spent on building potential structures that are torn down again and again after the external funding ends.

This is not only our impression but also an experience that we share with many colleagues from HEIs all over Germany who we are in regular exchange with. The implementation of health-promoting projects, as successful as they may have been, into the institutional structures of HEIs is often failing. Projects funded as part of the Prevention Act in the context of HEIs are at risk of expiring after the funding period if again individual researchers themselves do not successfully acquire additional (and again timely limited) third-party funding. In our opinion, the actual idea of the Prevention Act fails here.

## 3. Conclusion

In an international comparison, up to now only a very small number of German HEIs have adopted the Okanagan Charter and embedded health into all aspects of campus culture, across the administration, operations, and academic mandates. In most cases, health promotion at HEIs does not go beyond project character. The German Prevention Act does not provide an adequate solution to this lack when it comes to the higher education system, as it does not guarantee sustainable funding of successfully developed health-promoting structures after the end of an initial funding period. Health promotion therefore still relies on the commitment of individual scientists and/or the goodwill of potent donators.

In the German higher education system, a lot is based on timely limited funding, including the employment of most scientists. This brings supposed flexibility, but also problems in many places, such as staff continuity. However, this principle reaches its limits particularly quickly when it comes to building and maintaining sustainable structures, as they would be required in the field of health promotion. While leading international universities and comparable institutions worldwide have understood and implemented health promotion as a veritable competitive advantage, a lot of money and energy is spent in Germany without creating sustainable results. To change that, a new way of thinking and action is needed:

1) A clear strategy and requirement from the responsible state governments to the HEIs and their leaders that sustainable healthy higher education and working conditions are the cornerstones of modern, attractive, and successful HEIs.
2) Adequate and sustainable funding of German HEIs by policymakers to reduce the dependence on third-party funding.
3) A clear strategy of HEI leaders and administrators to recognize health as an elementary component of the culture at HEIs and to provide this goal with appropriate and sustainable resources.
4) It is our hope that the great opportunities offered by systemic health promotion in HEIs will also be recognized in Germany so that the system remains successful, attractive, and competitive.

## Author contributions

All authors listed have made a substantial, direct, and intellectual contribution to the work and approved it for publication.

## Footnotes

^1^A lifeworld (German: “Lebenswelt”) may be understood as a world that is self-evident or given and in which subjects spend most of their daily time (e.g. work, school, university, kindergarten, and home). A lifeworld has relatively constant but influenceable conditions, which can be, according to a health promoting approach, designed or influenced to promote health.

^2^What qualifies the authors as experts for the present topic? PD and MS are senior scholars at the University of Mainz (JGU), the biggest HEI in Rhineland-Palatinate, Germany, comprising 10 faculties, the School of Music Mainz, the School of Art Mainz, and the International Preparatory and Language Center. The JGU is the home of 76 disciplines and the lifeworld of around 31,000 students (4). Supported with financial resources of a statutory health insurance company in the framework of the German Prevention Act, the Healthy Campus Mainz project was initiated at the JGU in 2018. This interdisciplinary research project aims to create, evaluate, and implement an evidence-based, sustainable, and holistic student health management program at the JGU (5). PD and MS have been part of the project team since the project started, with PD in the leading role. In the course of their work, they have experienced the chances and opportunities as well as the obstacles and pitfalls that such a project entails in the context of a German university setting. In summer 2022, PD and MS were responsible for the organization of an international conference on health promotion in university students that took place at the JGU.
